# Whole-Genome Insights into the Genetic Basis of Conformation Traits in German Black Pied (DSN) Cattle

**DOI:** 10.3390/genes16040445

**Published:** 2025-04-10

**Authors:** Amelie Mandel, Monika Reißmann, Gudrun A. Brockmann, Paula Korkuć

**Affiliations:** 1 Albrecht Daniel Thaer-Institute for Agricultural and Horticultural Sciences, Animal Breeding and Molecular Genetics, Humboldt-Universität zu Berlin, Invalidenstr. 42, 10115 Berlin, Germany; amelie-mandel@gmx.de (A.M.); monika.reissmann@hu-berlin.de (M.R.); gudrun.brockmann@hu-berlin.de (G.A.B.); 2 Leibniz Institute for Zoo and Wildlife Research, Alfred-Kowalke-Straße 17, 10315 Berlin, Germany

**Keywords:** genome-wide association study (GWAS), endangered cattle breed, dual-purpose cattle, whole-genome sequencing (WGS), conservation

## Abstract

Background: The German Black Pied Dairy (DSN) cattle is an endangered dual-purpose breed considered an ancestor of the modern Holstein population. DSN is known for its high milk yield, favorable milk composition, and good meat quality. Maintaining a functional body structure is essential for ensuring sustained performance across multiple lactations in dual-purpose breeds like DSN. This study aims to identify candidate genes and genetic regions associated with conformation traits in DSN cattle through genome-wide association studies (GWAS). Methods: The analysis utilized imputed whole-genome sequencing data of 1852 DSN cows with conformation data for 19 linear traits and four composite scores derived from these traits. GWAS was performed using linear mixed models. Results: In total, we identified 118 sequence variants distributed across 24 quantitative trait locus (QTL) regions comprising 74 positional candidate genes. Among the most significant findings were variants associated with “Rump width” on chromosome 21 and “Teat length” on chromosome 22, with *AGBL1* and *SRGAP3* identified as the most likely candidate genes. Additionally, a QTL region on chromosome 15 linked to “Central ligament” contained 39 olfactory receptor genes, and a QTL region on chromosome 23 associated with “Hock quality” included eight immune-related genes, notably, BOLA and TRIM family members. Conclusions: Selective breeding for favorable alleles of the investigated conformation traits may contribute to DSN’s longevity, robustness, and overall resilience. Hence, continuous focus on healthy udders, feet, and legs in herd management contributes to preserving DSN’s positive traits while improving conformation.

## 1. Introduction

German Black Pied Cattle (DSN, German: Deutsches Schwarzbuntes Niederungsrind) originated from the coastal grasslands along the North Sea in Denmark, northern Germany, and the Netherlands. Today, DSN represents an endangered dual-purpose cattle breed with a small population size (2514 cows and 27 bulls as of 2021) [1]. Despite a relatively limited effective population size (Ne = 106.9), DSN exhibits substantial genetic milk yield potential, ranging from 7000 to over 8000 kg at 4.3% fat and 3.7% protein, and maintains desirable conformation traits, including a harmonious body structure, sturdy hooves, and a generally horned and black-and-white coat pattern [2,3].

Previous genome-wide association studies (GWAS) have investigated conformation traits in various cattle populations, including Chinese Holstein, Fleckvieh, Swiss Brown, and mixed Nordic dairy breeds, uncovering multiple QTL regions and candidate genes related to dairy character, udder conformation, feet and legs, and overall body structure [4,5,6,7]. These studies have identified genes implicated in cellular structure, metabolic pathways, immune responses, mammary gland development, and musculoskeletal integrity. Some candidate genes, such as those associated with dairy character or udder traits, appear to overlap across multiple breeds, while others exhibit breed-specific associations. These patterns reflect the genetic complexity underlying conformation traits and highlight the importance of breed-specific analyses, larger sample sizes, and functional validation to improve health, productivity, and sustainability in diverse cattle populations.

Within the DSN breed, previous GWAS have targeted traits such as milk production, mastitis, fertility, and resilience against endoparasites [8,9,10,11,12]. For instance, the favorable variant of the K232A polymorphism in *DGAT1* (diacylglycerol O-acyltransferase 1) is already fixed in DSN, and therefore, selection for the favorable *DGAT1* variant cannot further enhance milk yield. Furthermore, the *MGST1* (microsomal glutathione-S-transferase 1) gene, as well as genes of the casein gene cluster, both previously identified in Holstein and other breeds, appear relevant for breed improvement in DSN. These studies demonstrate the value of GWAS in uncovering genetic factors that may drive productivity and adaptability, even in endangered and relatively small cattle populations.

The primary goal of this study is to identify genomic regions and candidate genes associated with conformation traits in DSN cattle using whole-genome sequencing (WGS) data. In Germany, DSN cattle are routinely evaluated by trained personnel for 19 linear conformation traits during their first lactation. Understanding the genomic basis of these traits and the composite scores derived from these traits will enable more targeted and effective selection strategies to improve the body conformation and, thereby, the productivity and health of the animals. Ultimately, such insights can provide actionable genetic markers to guide breeding decisions to enhance the sustainability, health, and overall performance of DSN cattle and ensure their continued competitiveness compared to larger commercial dairy populations.

## 2. Materials and Methods

### 2.1. Population and Phenotypes

Conformation data of 1852 DSN cows with available genotypes, born between 2005 and 2018, were obtained from the breeding company RBB Rinderproduktion Berlin-Brandenburg GmbH (Groß Kreutz, Germany) as of April 2020. Only farms with at least 20 DSN cows were considered to minimize farm-related environmental influences in the GWAS. The conformation data consisted of 19 linear (LIN) traits contributing to the four composite (COM) scores “Dairy strength”, “Body conformation”, “Legs&Feet”, and “Mammary”, as listed in Table 1. The linear traits describe the external characteristic of each cow assessed once during the first lactation using a classification scheme by a trained member of the German Federal Association of Swine and Cattle (German: Bundesverband Schwein und Rind). All linear traits were measured on a scale from 1 to 9, except for “Stature” (LIN_STATURE_) which was measured in centimeters (cm). The composite scores, which were derived from the linear traits, are based on a 100-point system, with values ranging from 65 to 88 (for cows in their first lactation, the maximum attainable score is 88). To determine potential relationships among the four composite scores and the individual linear traits, pairwise correlations were calculated using the cor.test() function in R.

### 2.2. Genotypes

Genotypes of the 1852 DSN cows were available from Illumina BovineSNP50 (Illumina Inc., San Diego, CA, USA), EuroG MD (EuroGenomics, The Hague, The Netherlands), custom DSN200K SNP chips (Thermo Fisher Scientific, Waltham, MA, USA), and whole-genome sequencing [10,13]. Genotypes were imputed to WGS data density using Beagle v5.1 [14]. Genotypes from the DSN200K chip were directly imputed to WGS data positions using a reference panel of 304 sequenced DSN cattle. Genotypes from Illumina BovineSNP50 and EuroGenomics EuroG MD chips were initially imputed to match the DSN200K chip positions using a reference panel of 1580 DSN cattle genotyped with the DSN200K chip, and subsequently imputed to WGS level. Variants with a minor allele frequency (MAF) of < 5% and a variant call rate of < 90% were removed with VCFtools v0.1.17 [15], resulting in 12,024,918 variants suitable for GWAS. The genomic positions of variants provided in this study are based on the *Bos taurus* genome version ARS-UCD1.2, serving as the reference genome for accurate alignment and annotation [16].

### 2.3. GWAS Model

For the continuously scaled values of the composite scores (COM_DAIRY STRENGTH_, COM_BODY CONFORMATION_, COM_LEGS&FEET_, COM_MAMMARY_), as well as for the linear trait LIN_STATURE_, GWAS were performed using mixed linear regression models using the basic lm() function in R. For the other 18 ordinally scaled linear traits, ordinal regression models using either the clm() function from the R package ordinal v2023.12-4.1 [17] or the polr() function from the R package MASS v7.3-58.2 [18] were used. While clm() was computationally faster, polr() performed better with small fixed-effect groups.

For each trait, the optimal model was determined using the Akaike Information Criterion (AIC) to evaluate whether the inclusion of additional fixed effects improved model fit. Population stratification (*ps*) was included as a mandatory fixed effect due to its known influence on GWAS results. Additional fixed effects were added one at a time and retained if their inclusion decreased AIC by at least 10 units (ΔAIC ≤ −10). Table S1 provides an overview of the AIC changes and the final model specifications for each trait. Depending on the trait, the final models included combinations of the following fixed effects: farm (*fa*), birth year (*by*), birth season (*bs*), calving year (*cy*), calving season (*cs*), age at first calving (*fc*), and day in lactation (*ld*). Genotype (*gt*) and residual error (*e*) were included in all models. The full model is expressed as follows:

(1) Y=ps+fa+by+bs+cy+cs+fc+ld+gt+e

Population stratification was evaluated using a concordance test in PLINK v1.9 [19] using a *p*-value of 0.0000001 identifying 46 family clusters. Birth years ranged from 2005 to 2018 and calving years between 2007 and 2020. To account for low sample sizes in certain categories, grouping was applied: birth years ≤ 2009 and ≥2017, and calving years ≤ 2011 and ≥2019. Days in lactation were categorized into five groups (30, 80, 120, 160, and ≥200). Genotype groups with fewer than 30 animals were excluded from GWAS to ensure stable effect estimation of statistical tests.

### 2.4. Significant Threshold and QTL Definition

*p*-values from the GWAS were adjusted for an inflation factor λ = 1.2 on the autosomal chromosomes and chromosome X, separately. Before correction, the λ values for the autosomal chromosomes ranged from 1.04 for LIN_FOOT_ANGLE_ to 2.43 for LIN_REAR_TEAT_, and for chromosome X from 1.08 for LIN_FOOT_ANGLE_ to 3.71 for LIN_REAR_TEAT_ (Table S2). To control the risk of false-positive associations arising from multiple testing, we adjusted the *p*-values using the Bonferroni correction method. The number of independent tests was estimated through linkage disequilibrium (LD)-based pruning in PLINK v1.9, applying an r^2^ threshold of 0.6, a window size of 2 Mb, and a step size of 100, resulting in 569,464 independent variants. The significance thresholds were calculated by dividing the chosen significance levels (highly significant: 0.01, significant: 0.05, and suggestive: 0.1) by the number of independent variants corresponding to the thresholds −log_10_(*p*) of 7.76, 7.06, and 6.76, respectively. The *p*-values between genotype groups in the variant effect plots were calculated using the Wilcoxon test. Regions with variants above the suggestive threshold within windows of 2.5 Mb were defined as QTL regions. Distances up- and downstream of the top variant were extended to 150 kb, respectively, if they were smaller than 150 kb.

### 2.5. QTL Annotation

The identified QTLs were further investigated for candidate genes and variants. Protein-coding genes were obtained from Ensembl release version 110 [20]. Candidate genes in each respective QTL region were analyzed for gene ontology (GO) term enrichment using g:profiler version e111_eg58_p18_30541362 [21]. The g:SCS thresholds with *p* < 0.05 for significant GO term enrichments were used. Variants within QTL regions were assessed for their impact consequence on gene transcripts using Ensembl Variant Effect Predictor (VEP) [22]. For the VEP investigation, rare variants with an MAF between 1–5% within the identified QTL regions were also considered. Due to the extensive amount of the VEP output, only variants exhibiting moderate or high impact on gene transcripts were retained. Further, the identified QTL regions were examined for overlap with previously published associations and QTLs with the trait categories “Production” or “Exterior” obtained from cattleQTLdb release 54 [23]. Using the R package easyPubMed version 2.13 [24], the corresponding PubMed IDs of publications were obtained. Analyses were conducted using statistical software R version 4.3.1 [25]. Additional R packages used for data preparation, analyses, and visualization were reshape2 v1.4.4, ggpubr v0.6.0, and ggplot2 v3.5.1 [26,27,28].

## 3. Results

### 3.1. Correlation Analysis

The values of the four composite scores COM_DAIRY STRENGTH_, COM_BODY CONFORMATION_, COM_LEGS&FEET_, and COM_MAMMARY_ were investigated for correlations with each other. The correlation between COM_BODY CONFORMATION_ and COM_DAIRY STRENGTH_ was moderately positive reaching *r* = 0.43. All other pairwise correlations were low and positive, ranging between *r* = 0.12 between COM_LEGS&FEET_ and COM_BODY CONFORMATION_ and *r* = 0.22 for COM_MAMMARY_ and COM_DAIRY STRENGTH_.

The pairwise correlation analysis between composite scores and linear traits revealed that, as expected, almost all linear traits belong to their respective composite score reaching moderate (*r* = 0.30) to high correlations (*r* = 0.83) (Table 2). For COM_DAIRY STRENGTH_, a moderate correlation was observed with LIN_DAIRY_CHARACTER_ (*r* = 0.54). Interestingly, a weak positive and negative correlation of COM_DAIRY STRENGTH_ was observed with the traits LIN_STATURE_ (*r* = 0.34) and LIN_BCS_ (*r* = −0.27), respectively, which are actually linear traits belonging to COM_BODY CONFORMATION_. For COM_BODY CONFORMATION_, moderate to high positive correlations with five out of six linear traits belonging to this composite score were observed, except for LIN_RUMP_ANGLE_, which had no correlation (*r* = −0.06). For COM_LEGS&FEET_, moderate to high positive correlations were observed with four out of five linear traits belonging to this composite score, but negative correlation with LIN_REAR_LEG_SIDE_ (*r* = −0.30). Additionally, a weak positive correlation of COM_LEGS&FEET_ with LIN_REAR_UDDER_ was observed (*r* = 0.25), a linear trait that actually belongs to COM_MAMMARY_. Lastly, COM_MAMMARY_ showed moderate to high correlation with six out of seven linear traits belonging to this composite score. LIN_TEAT_LENGTH_ was not correlated with COM_MAMMARY_ (*r* = −0.01), although it contributed to this composite score.

### 3.2. Overview of GWAS Results for All Composite Scores and Linear Traits

After the correction of *p*-values for inflation factor λ and Bonferroni, 118 sequence variants above the suggestive threshold (*p* < 0.1) were identified across 19 chromosomes for 14 conformation traits (Table S3). Among these, six variants were highly significant (*p* < 0.01) for two traits (LIN_RUMP_WIDTH_ and LIN_TEAT_LENGTH_), and 25 variants were significant (*p* < 0.05) for twelve traits or composite scores (COM_DAIRY STRENGTH_, LIN_DAIRY_CHARACTER_, COM_BODY CONFORMATION_, LIN_BCS_, LIN_RUMP_ANGLE_, LIN_RUMP_WIDTH_, COM_LEGS&FEET_, LIN_CENTRAL_LIGAMENT_, LIN_FORE_UDDER_, LIN_LOCOMOTION_, LIN_REAR_LEG_SIDE_, and LIN_REAR_UDDER_). The 118 variants could be grouped into 24 QTLs, each represented by its top variant (Table 3). In the identified QTLs, 74 positional candidate genes (Table 4) and 451 unique variants with moderate to high impacts on gene transcripts were identified (Table S4). Below, we provide detailed descriptions of exemplary QTL regions that were highly significant (*p* < 0.01) or had additional insights available, including information on moderate- and high-impact variants, interesting candidate genes, significant GO term enrichment (Table S5), and/or overlap with previously reported associations from cattleQTLdb (Table S6).

### 3.3. DAIRY STRENGTH—Association for “Dairy Character”

**Figure 1:**
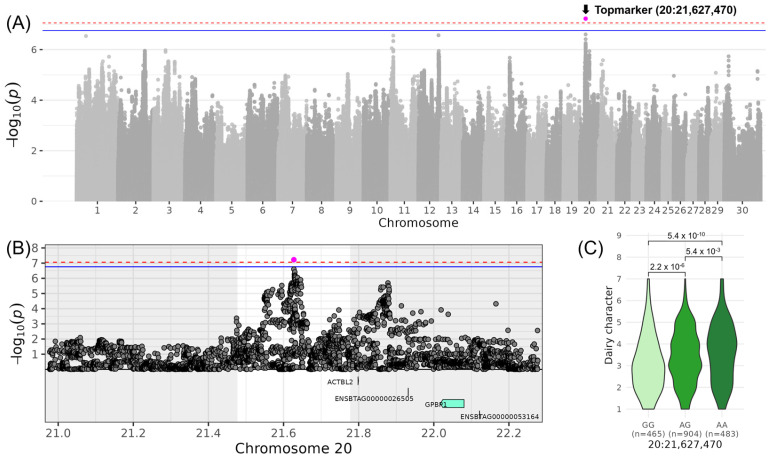
Genome-wide association results for LIN_DAIRY_CHARACTER_ on chromosome 20 in DSN cattle. (**A**) Manhattan plot displaying the genomic positions versus the significance of genetic variants. The horizontal lines represent Bonferroni-corrected thresholds for genome-wide significance (red, *p* < 0.05) and suggestive association (blue, *p* < 0.1). (**B**) Regional association plot highlighting a detailed region around the significant locus, showing positional candidate genes. (**C**) Variant effect plot illustrating genotype-specific phenotypic effects for the top variant rs381893684 at position 21,627,470 bp. Additionally, *p*-values between genotype groups and the number of animals in each genotype group are provided.

For LIN_DAIRY_CHARACTER_, which contributes to the composite score COM_DAIRY STRENGTH_, a significant locus on chromosome 20 was identified with the top variant rs381893684 located at 21,627,470 bp (−log_10_(*p*) = 7.23, Table 3, Figure 1). In the DSN population, the allele G and the allele A occurred both with a frequency of 50%. The β-effect of 0.43 indicated that DSN cows with the homozygous genotype AA scored 0.86 higher with respect to LIN_DAIRY_CHARACTER_ compared to DSN cows with the homozygous genotype GG. The corresponding QTL ranged from 21,477,400 to 21,777,400 bp and did not contain any positional candidate gene (Table 4). Around 250 kb downstream of the top variant rs381893684 is a second non-significant peak at 21,877,877 bp. The two top variants of the adjacent peaks were in low linkage (*r*^2^ = 0.35). Between those two peaks, the gene *ACTBL2* (Actin β like 2) was located.

### 3.4. BODY CONFORMATION—Associations for “Rump Width” and “Rump Angle”

**Figure 2:**
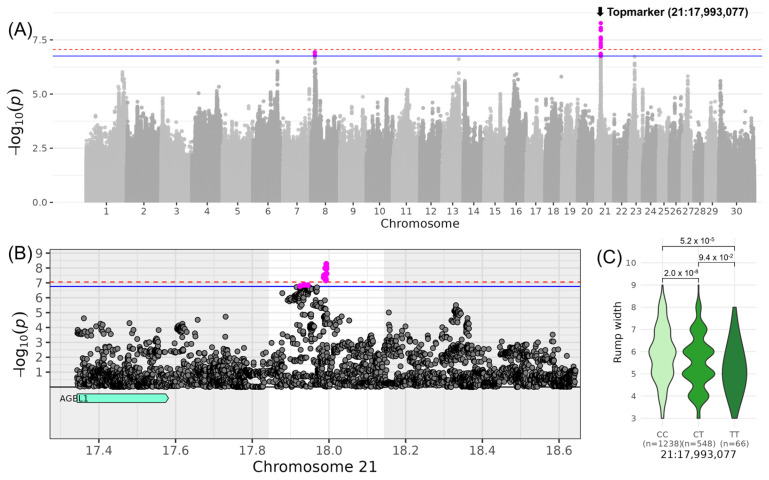
Genome-wide association results for LIN_RUMP_WIDTH_ on chromosome 21. (**A**) Manhattan plot displaying the genomic positions versus the significance of genetic variants. The horizontal lines represent Bonferroni-corrected thresholds for genome-wide significance (red, *p* < 0.05) and suggestive association (blue, *p* < 0.1). (**B**) Regional association plot highlighting a detailed region around the significant locus, showing positional candidate genes. (**C**) Variant effect plot illustrating genotype-specific phenotypic effects for the top variant rs210390204 at 17,993,077 bp. Additionally, *p*-values between genotype groups and the number of animals in each genotype group are provided.

For LIN_RUMP_WIDTH_, which belongs to the composite score COM_BODY CONFORMATION_, highly significant variants were identified on chromosome 21. The top variant rs210390204 was located at 17,993,077 bp (−log_10_(*p*) = 8.27, Table 3, Figure 2). The minor allele T of this variant occurred with a frequency of 19% in the examined DSN population. DSN cows with the homozygous genotype TT scored −1.16 lower in the linear description for LIN_RUMP_WIDTH_ compared to cows with the homozygous genotype CC (β_MA_ = −0.58). In the respective QTL region ranging from 17,843,077 to 18,143,077 bp, no candidate genes were identified. The nearest gene *AGBL1* (AGBL carboxypeptidase 1) was located approximately 261 kb upstream of the QTL region.

**Figure 3:**
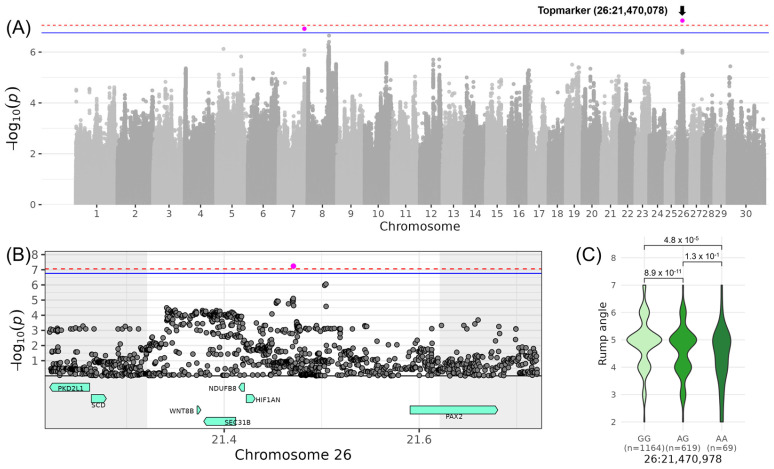
Genome-wide association results for LIN_RUMP_ANGLE_ on chromosome 26. (**A**) Manhattan plot displaying the genomic positions versus the significance of genetic variants. The horizontal lines represent Bonferroni-corrected thresholds for genome-wide significance (red, *p* < 0.05) and suggestive association (blue, *p* < 0.1). (**B**) Regional association plot highlighting a detailed region around the significant locus, showing positional candidate genes. (**C**) Variant effect plot illustrating genotype-specific phenotypic effects for the top variant rs110956215 at 21,470,978 bp. Additionally, *p*-values between genotype groups and the number of animals in each genotype group are provided.

For LIN_RUMP_ANGLE_, which is another trait contributing to COM_BODY CONFORMATION_, significance was reached on chromosome 26. The top variant rs110956215 was located at 21,470,978 bp (−log_10_(*p*) = 7.24), and its minor allele A had a frequency of 21% and reduced LIN_RUMP_ANGLE_ by 0.55 points (Table 3, Figure 3). In the corresponding QTL region, ranging from 21,320,978 to 21,620,978 bp, the five candidate genes *SEC31B* (SEC31 homolog B, COPII coat complex component), *NDUFB8* (NADH:ubiquinone oxidoreductase subunit B8), *HIF1AN* (Hypoxia inducible factor 1 subunit α inhibitor), *WNT8B* (Wnt family member 8B), and *PAX2* (Paired box 2) were located (Table 4). In *SEC31B*, four tolerated missense variants (26:21381141; 26:21,392,002; 26:21,393,52; 26:21,395,038) and one deleterious missense variant (26:21,388,919) were located. The deleterious variant was found in exon 19 out of 26 exons and changes the amino acid from valine to methionine at the protein position 803 out of 1122 amino acids not affecting any protein domain (Table S4). In *HIF1AN*, a low confidence tolerated missense variant (26:21,422,781) was found changing alanine to proline at protein position 12 out of 349 amino acids located in a disordered region (www.uniprot.org, accessed on 5 February 2025). In *PAX2*, an 8 bp indel (26:21,591,127–21,591,134) causes a frameshift at positions 24–26 of the 489 amino acid sequence, resulting in a high-impact alteration on the respective gene transcript and encoded protein. Furthermore, GO term enrichment revealed the significant GO term “[protein]-asparagine 3-dioxygenase activity” (GO:0036140) involving *HIF1AN* (Table S5).

### 3.5. LEGS&FEET—Associations for “Rear Leg Side” and “Hock Quality”

**Figure 4:**
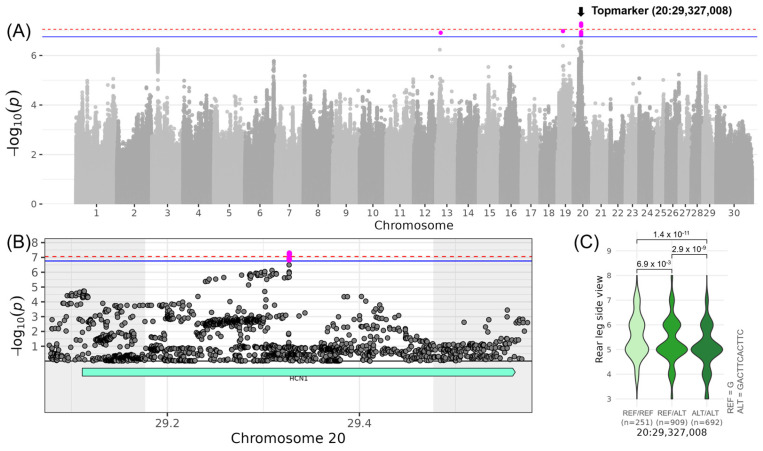
Genome-wide association results for LIN_REAR_LEG_SIDE_ on chromosome 20. (**A**) Manhattan plot displaying the genomic positions versus the significance of genetic variants. The horizontal lines represent Bonferroni-corrected thresholds for genome-wide significance (red, *p* < 0.05) and suggestive association (blue, *p* < 0.1). (**B**) Regional association plot highlighting a detailed region around the significant locus, showing positional candidate genes. (**C**) Variant effect plot illustrating genotype-specific phenotypic effects for the top variant at 29,327,008 bp. Additionally, *p*-values between genotype groups and the number of animals in each genotype group are provided.

With regard to the linear traits contributing to the composite score COM_LEGS&FEET_, the two most interesting loci were found on chromosomes 20 and 23. On chromosome 20, the region was associated with LIN_REAR_LEG_SIDE_ with the top variant (no rs-ID) located at 29,327,008 bp (−log_10_(*p*) = 7.29) (Table 3, Figure 4). The minor allele G of the top variant occurred with a frequency of 38% and increased LIN_REAR_LEG_SIDE_ by 0.44. The QTL region spanned the genomic coordinates from 29,177,008 to 29,477,008 bp encompassing the whole gene *HCN1* (hyperpolarization-activated cyclic nucleotide-gated potassium channel 1) with the top variant in the middle of the gene (Table 4). No sequence variants of moderate or high impact were found in this QTL region, but significant GO term enrichments. Involving *HCN1*, the GO terms “HCN channel complex” (GO:0098855) and “intracellular cAMP-activated cation channel activity involved in the regulation of presynaptic membrane potential” (GO:0140232), and the Reactome pathway “HCN channels” (REAC:R-BTA-1296061) were found significant (Table S5).

The above-identified locus for LIN_DAIRY_CHARACTER_ (on chromosome 20 at 21.8 Mb) is around 7.5 Mb upstream of the locus for LIN_REAR_LEG_SIDE_ (on chromosome 20 at 29.3 Mb). Despite the proximity, the top variants of those two QTL regions were not in linkage (*r*^2^ = 0.07).

**Figure 5:**
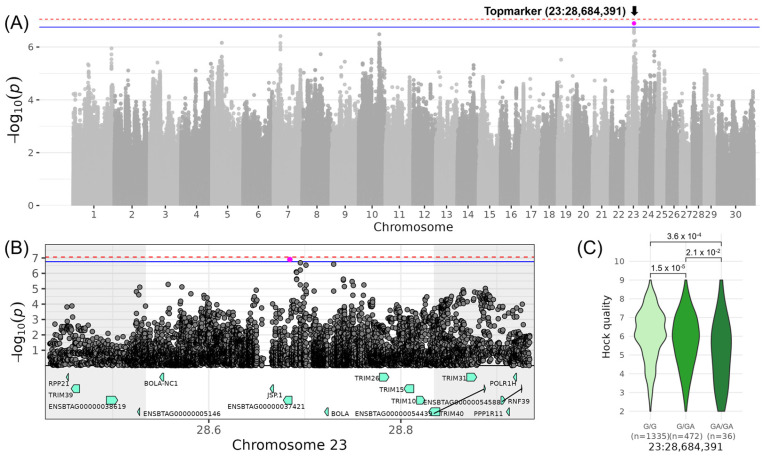
Genome-wide association results for LIN_HOCK_QUALITY_ on chromosome 23. (**A**) Manhattan plot displaying the genomic positions versus the significance of genetic variants. The horizontal lines represent Bonferroni-corrected thresholds for genome-wide significance (red, *p* < 0.05) and suggestive association (blue, *p* < 0.1). (**B**) Regional association plot highlighting a detailed region around the significant locus, showing positional candidate genes. (**C**) Variant effect plot illustrating genotype-specific phenotypic effects for the top variant rs800639948 at 28,684,391 bp. Additionally, *p*-values between genotype groups and the number of animals in each genotype group are provided.

On chromosome 23, a QTL for LIN_HOCK_QUALITY_ with the top variant rs800639948 located at 28,684,391 bp was identified (−log_10_(*p*) = 6.90, Table 3, Figure 5). The minor allele of the indel GA was observed with a frequency of 15% in the examined DSN population and reduced LIN_HOCK_QUALITY_ by 0.52, indicating a decrease of −1.04 in the score for LIN_HOCK_QUALITY_ for DSN cows with the homozygous genotype GA/GA. The corresponding QTL spanning the genomic region from 28,534,391 to 28,834,391 bp contained eight candidate genes: *BOLA* (MHC class I heavy chain), *TRIM10* (tripartite motif containing 10), *TRIM15* (tripartite motif containing 15), *BOLA-NC1* (non-classical MHC class I antigen), *JSP.1* (MHC Class I JSP.1), *TRIM26* (tripartite motif containing 26), *TRIM40* (tripartite motif containing 40), and ENSBTAG00000037421, which is a novel gene coding for a *TRIM26*-like protein (Table 4). The top variant was located within intron 1 of ENSBTAG00000037421, which contains a total of four introns. This QTL region shows also high variability containing 150 variants with moderate impact (107 tolerated and 43 deleterious missense variants) and 21 with high impact on gene transcripts referring to frameshift, splice acceptor or donor, start lost, stop gained, and stop lost variants (Table S4). GO enrichment analysis of the candidate genes within this QTL revealed significant associations with the biological process of “immune response” (GO:0006955) attributed to the genes *BOLA*, *TRIM10*, *TRIM15*, *JSP.1*, *TRIM26*, and *TRIM40* (Table S5). In particular, the genes *BOLA* and *JSP.1* are included, e.g., in the GO terms “antigen processing and presentation of endogenous peptide antigen via MHC class I via ER pathway” (GO:0002484) and “positive regulation of T cell mediated cytotoxicity” (GO:0001916), and the TRIM genes, e.g., in “negative regulation of viral life cycle” (GO:1903901) and “ubiquitin protein ligase activity” (GO:0061630).

### 3.6. MAMMARY—Associations for “Central Ligament” and “Teat Length”

**Figure 6:**
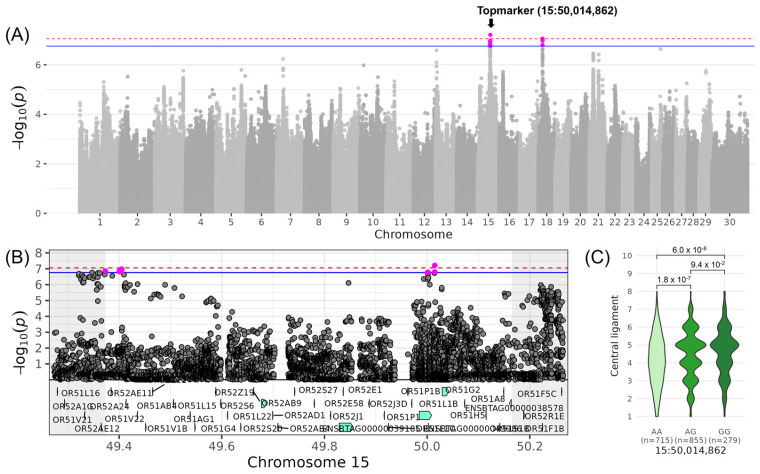
Genome-wide association results for LIN_CENTRAL_LIGAMENT_ on chromosome 15. (**A**) Manhattan plot displaying the genomic positions versus the significance of genetic variants. The horizontal lines represent Bonferroni-corrected thresholds for genome-wide significance (red, *p* < 0.05) and suggestive association (blue, *p* < 0.1). (**B**) Regional association plot highlighting a detailed region around the significant locus, showing positional candidate genes. (**C**) Variant effect plot illustrating genotype-specific phenotypic effects for the top variant at 50,014,862 bp. Additionally, *p*-values between genotype groups and the number of animals in each genotype group are provided.

The QTL with the highest gene density was found for LIN_CENTRAL_LIGAMENT_, a trait belonging to the composite score COM_MAMMARY_, on chromosome 15 (Table 3, Figure 6). This QTL ranged from 49,373,191 to 50,164,862 bp and was represented by the intergenic top variant at 50,014,862 bp (−log_10_(*p*) = 7.21). The minor allele G of this variant occurred with a frequency of 38% and increased LIN_CENTRAL_LIGAMENT_ by 0.38. The 34 genes in this QTL region belonged either to the *OR51* (olfactory receptor family 51) or *OR52* (olfactory receptor family 52) gene family or were novel (Table 4). The 34 genes contained altogether 12 variants with high and 186 variants with moderate impact on gene transcripts (Table S4). The high-impact variants were frameshift and stop-gained mutations located mainly within the *OR51* and *OR52* genes. For example, in *OR51AG1*, insertions at positions 49,538,343 bp and 49,538,344 bp resulted in a frameshift mutation at position 111 out of 315 amino acids of the protein. For *OR51L15*, another frameshift variant was located at positions 49,597,808 bp and 49,597,809 bp, resulting in a deletion at positions 133–134 out of 319 amino acids of the protein. GO term enrichment analysis revealed significant assignment to e.g., “detection of chemical stimulus involved in sensory perception of smell” (GO:0050911) and “sensory perception of smell” (GO:0007608) (Table S5).

**Figure 7:**
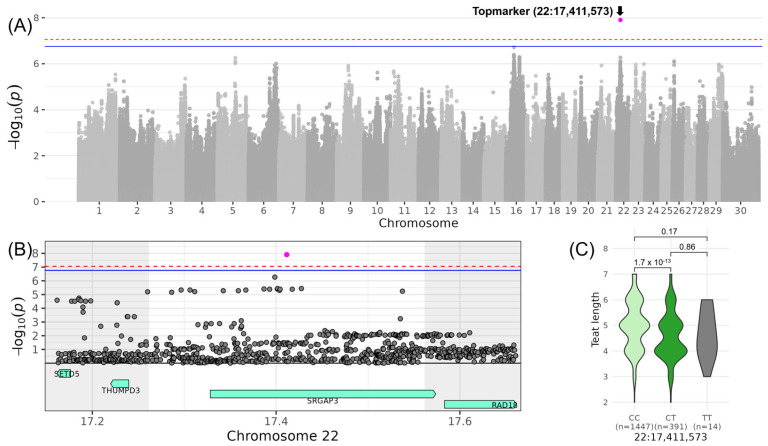
Genome-wide association results for LIN_TEAT_LENGTH_ on chromosome 22. (**A**) Manhattan plot displaying the genomic positions versus the significance of genetic variants. The horizontal lines represent Bonferroni-corrected thresholds for genome-wide significance (red, *p* < 0.05) and suggestive association (blue, *p* < 0.1). (**B**) Regional association plot highlighting a detailed region around the significant locus, showing positional candidate genes. (**C**) Variant effect plot illustrating genotype-specific phenotypic effects for the top variant rs378674848 at 17,411,573 bp. Additionally, *p*-values between genotype groups and the number of animals in each genotype group are provided.

The most significant association for a trait belonging to COM_MAMMARY_ was identified on chromosome 22 for LIN_TEAT_LENGTH_, with the top variant rs378674848 located at 17,411,573 bp (−log_10_(*p*) = 7.91, Table 3, Figure 7). The minor allele T of this variant occurred with a frequency of 10% in the investigated DSN population. DSN cows with the heterozygous genotype CT had a reduced LIN_TEAT_LENGTH_ by 0.78 in comparison to cows with the homozygous CC genotype. Within the QTL ranging from 17,261,573 to 17,561,573 bp, the top variant was located in the intron of the candidate gene *SRGAP3* (SLIT-ROBO Rho GTPase activating protein 3) (Table 4).

## 4. Discussion

In this study, we explored 19 linear conformation traits and four composite scores derived from these traits in 1852 DSN cattle using GWAS with approximately 12 million sequence variants. Despite limitations imposed by the relatively small DSN population, we identified 118 associated sequence variants mapped to 24 QTLs. These findings highlight the complexity of the genetic architecture underlying conformation traits and underscore the importance of expanding sample sizes in future research.

Our correlation analyses revealed generally low positive pairwise correlations among the four composite scores, supporting their utility as distinct metrics for assessing various aspects of conformation. The moderate correlation between COM_BODY CONFORMATION_ and COM_DAIRY STRENGTH_ suggests a partially shared genetic and/or phenotypic basis, without replacing each other. Examining the linear traits within their respective composite scores showed that most linear traits were appropriately assigned. Exceptions, such as LIN_RUMP_ANGLE_ and LIN_TEAT_LENGTH,_ which had no correlation to their own or any of the other composite scores, may represent unique trait dimensions valuable for specialized selection strategies. Furthermore, negative correlations between LIN_REAR_LEG_SIDE_ and COM_LEGS&FEET_ as well as between LIN_BCS_ and COM_DAIRY STRENGTH_ underscore potential trade-offs. The latter correlation aligns with earlier findings of an unfavorable genetic correlation between body condition score and milk yield, emphasizing the need to balance productivity with health and longevity in breeding programs [29]. Similarly, Khmelnychyii et al. (2023) found a negative correlation between LIN_BCS_ and COM_DAIRY STRENGTH_ of *r* = −0.47 in the Ukrainian Brown dairy breed [30]. This suggests that animals selected for high milk production (COM_DAIRY STRENGTH_) might have lower body condition scores (LIN_BCS_), emphasizing the need to ensure that high milk production does not adversely affect body condition to maintain health and longevity or vice versa. Further, Khmelnychyii et al. (2023) reported a positive correlation of *r* = 0.33 between “Rear Leg Set” (a combination of “Rear Leg Side View” and “Rear Leg Rear View”) and COM_LEGS&FEET_ [30], while, in that study, the correlation of COM_LEGS&FEET_ with LIN_REAR_LEG_SIDE_ was negative (*r* = −0.30), but with LIN_REAR_LEG_REAR_ positive (*r* = 0.61). This divergence suggests that these two linear traits contribute differently to the composite score COM_LEGS&FEET_ in those breeds, yet the overall results may still align with the reported finding. Overall, correlation coefficients between these traits are valuable for validating the alignment of linear traits with composite scores and assessing whether adjustments or a new classification system may be needed.

The limited population size of DSN cattle, together with complex family structures, contributed to the elevated *p*-value inflation observed across nearly all conformation traits. In contrast to previously investigated health, fertility, and milk production traits in DSN [8,10,11], the inflation factors reported in this study were notably higher. This suggests additional underlying factors beyond population size and structure alone. For example, the composite scores, which integrate multiple linear traits, may inherently increase variability and thus inflate test statistics. Furthermore, the subjective nature of trait assessment (where trained classifiers apply standardized, yet potentially interpretable criteria) could introduce discrepancies. Such population-specific and methodological nuances highlight the need for careful adjustment and stringent quality control measures. In this study, *p*-values were adjusted for inflation, and population stratification was considered as a fixed effect in the statistical model, ensuring more reliable and robust GWAS results.

Several genomic regions identified in our study showed overlaps with previously reported QTLs for related traits in other cattle populations (Table S6). Notably, regions affecting “Stature” (LIN_STATURE_) on chromosomes 2 and 7 have been previously identified in Holstein, Montbéliarde, Simmental, and multiple other breeds [31,32]. For instance, the locus associated with “Rump width” (LIN_RUMP_WIDTH_) on chromosome 21 overlaps with genomic regions previously linked to economically relevant traits such as “Net merit” and “Length of productive life” in Holstein cattle [33]. The QTL we identified for the trait “Rump angle” (LIN_RUMP_ANGLE_) on chromosome 26 aligns with a previously described locus associated with “Residual feed intake”, suggesting potential biological interactions between structural conformation and feed efficiency [34]. The locus on chromosome 20 linked to “Rear leg side view” (LIN_REAR_LEG_SIDE_) overlaps with regions previously associated with “Body weight” and growth traits across multiple beef breeds, including Charolais, Hereford, and Simmental [35,36]. Furthermore, the locus influencing “Hock quality” (LIN_HOCK_QUALITY_) on chromosome 23 coincides with a known QTL for “Feet and leg conformation” in Holstein cattle, supporting its functional relevance for structural integrity [37]. These overlaps support the validity of our findings and reinforce the potential value of these genomic regions for targeted breeding strategies in DSN cattle.

Our identification of QTL regions harboring candidate genes related to cellular structure, immune responses, and developmental pathways provides insight into the genetic basis of conformation traits, reinforcing and extending earlier QTL findings.

For the trait LIN_DAIRY CHARACTER_, which contributes to the composite score COM_DAIRY STRENGTH_, a QTL region identified on chromosome 20 at 21.6 Mb did not contain any annotated genes. However, the closest gene, *ACTBL2*, encodes a highly conserved protein involved in cell motility and is expressed in all eukaryotic cells (www.uniprot.org, accessed on 5 February 2025). Although not directly linked to dairy traits, its fundamental role in cellular processes may indirectly influence overall health and performance.

Regarding the traits contributing to the composite score COM_BODY CONFORMATION_, QTLs were described for LIN_RUMP_WIDTH_ on chromosome 21 and LIN_RUMP_ANGLE_ on chromosome 26. On chromosome 21, no gene was within the QTL boundaries, but the nearest gene, *AGBL1*, encodes an enzyme involved in protein modification and function. It has been associated with longevity, productive lifespan, and disease susceptibility in cattle [38]. This region was also previously linked to traits such as “Teat length”, “Length of productive life”, and fertility traits like “Calving ease” and “Pregnancy rate” in Holstein cattle [33]. Such relationships support the idea that wider rumps may facilitate easier calving, thus linking conformation to fertility and longevity. On chromosome 26, five candidate genes were identified, including *PAX2*, *WNT8B*, and *HIF1AN*, all of which are involved in key developmental and physiological processes (www.proteinatlas.org, accessed on 5 February 2025). *PAX2*, a transcription factor, plays a critical role in kidney cell differentiation and the development of the urogenital tract, eyes, and central nervous system. *WNT8B* is involved in the development and differentiation of specific forebrain structures, including the hippocampal region. *HIF1AN* contributes indirectly to developmental processes by regulating cellular responses to oxygen availability, emphasizing its role in tissue adaptation and survival under hypoxic conditions. Additionally, this QTL has been associated with “Residual feed intake” in Holstein cattle [34], underscoring the potential pleiotropic influence of these genes on growth, feed efficiency, and structural traits.

Regarding the traits contributing to the composite score COM_LEGS&FEET_, QTLs were described for LIN_REAR_LEG_SIDE_ on chromosome 20 and LIN_HOCK_QUALITY_ on chromosome 23. The QTL on chromosome 20 at 29.3 Mb includes *HCN1*, an ion channel gene important in heart and nerve cells, which has been previously implicated in breast cancer [39]. This region has also been linked to “Metabolic body weight”, “Average daily gain”, and “Body weight” [35,36]. These associations suggest that cattle with favorable alleles in this region may exhibit enhanced feed efficiency and weight gain, supported by better overall health, including healthy legs. On chromosome 23, the QTL encompassing eight immune-related candidate genes was identified. The genes include BOLA and TRIM family members, which are integral to adaptive and innate immune responses in cattle [40,41,42]. Additionally, this region has been associated with “Feet and leg conformation” in Holstein cattle [37]. The immune function genes could contribute to better feet and leg health, establishing a connection to the LIN_HOCK_QUALITY_ trait and emphasizing the importance of immune function in maintaining structural soundness and longevity.

The following QTLs contributing to the composite score COM_MAMMARY_ were described: one affecting LIN_CENTRAL_LIGAMENT_ on chromosome 15 and another influencing LIN_TEAT_LENGTH_ on chromosome 22. The QTL on chromosome 15 contained 39 olfactory receptor genes, which are involved in olfactory and G protein-coupled receptor activities, transmembrane signaling, and sensory perception. Their expression in various tissues and organs, such as the olfactory epithelium of the nasal cavity [43], liver [44], lung [45], and pancreas [46], suggests a broader physiological role that could impact mammary function and growth. Moreover, this QTL region has been linked to “Body weight gain” in diverse breeds [35], potentially indicating pleiotropic effects on both growth and mammary development. On chromosome 22, the only candidate gene was *SRGAP3* encoding a GTPase-activating protein. No moderate- or high-impact variants or GO terms were assigned to this QTL, leaving its functional relevance open for future investigation.

The availability of whole-genome sequencing data of all individuals, which were either sequenced directly or imputed, made the search for causal sequence variants feasible. Some of the top variants were located directly within the coding sequences for proteins. Many of the top variants were located in deserts without genes. Those sequence variants could be a valuable hint on regulatory functions of the causal mutation rather than a distortion of the function of encoded proteins itself. Providing evidence for regulatory effects on gene functions requires additional more comprehensive analysis methods on the molecular level of gene regulation. Evidence for strong effects on gene function through regulatory variants has been shown repeatedly in diverse species [47]. Beyond the identified candidate genes, non-coding genes, regulatory mutations, and structural variants likely play a role in conformation traits but were not fully captured in this study. Non-coding RNAs and microRNAs regulate gene expression and could impact phenotypes without altering coding sequences [48]. Regulatory mutations in enhancers and promoters may influence gene activity, contributing to trait variability. Structural variants can disrupt genes or modify regulatory landscapes, affecting conformation. These elements require further investigation using long-read sequencing and functional genomics to fully understand the impact of genetic variation underlying the examined phenotypes.

The identification of these candidate genes across multiple chromosomes emphasizes the complex genetic architecture underlying conformation traits in cattle. Further research is needed to clarify the specific functions of these genes, particularly with respect to udder conformation and functionality. Such insights could not only advance our understanding of the genetic mechanisms at play but also enhance genetic selection strategies aimed at improving both dairy and dual-purpose cattle breeds. Increasing the sample size in future studies will be crucial for validating these findings and refining their applications in breeding programs.

## 5. Conclusions

The genetic architecture of conformation traits in DSN cattle is complex, involving multiple QTLs, each potentially influencing a range of structural, physiological, and developmental pathways. Correlation analyses between composite scores and linear conformation traits confirmed that most linear traits were appropriately assigned to their composite scores. However, certain linear traits showed weak or no correlation to their composite scores, suggesting unique trait dimensions that could inform more targeted selection strategies. The candidate genes identified within the QTL regions highlight a wide array of biological pathways influencing conformation traits, including those associated with cellular structure, immune function, and development. These insights open up promising avenues for future functional studies aimed at improving conformation traits in DSN cattle. In practical breeding, selecting animals that exhibit favorable linear traits, particularly “Rear leg side view” and “Hock quality”, as well as “Central ligament” and “Teat length”, could enhance the overall robustness and competitiveness of the DSN breed. However, attention must be given to the negative correlation between traits, ensuring that improvements in one trait do not come at the expense of another. By combining selective breeding with sound management practices focused on maintaining healthy udders and legs, breeders can preserve the positive attributes of the DSN while striving for advancements in conformation and productivity. Further research, including expanded sample sizes, functional validation of candidate genes, and the identification of causal mechanisms leading to functional changes of gene effects, is essential for refining genetic selection strategies.

## Figures and Tables

**Table 1:** Overview of the four composite (COM) scores and 19 linear (LIN) traits they consist of in DSN cows. The traits and scores are given with their description, the measurement unit and range, and the calculated minimum (min), mean, standard deviation (sd), and maximum (max) values in the investigated DSN cow population.

Trait	Description	Unit, Range	Min	Mean	Sd	Max
COM_DAIRY STRENGTH_	Composite score based on linear trait below	points, 65–88	70	79.1	3.0	86
LIN_DAIRY_CHARACTER_	Rip angle and openness, and bone flatness (angularity)	scale, 1–9	1	3.4	1.3	7
COM_BODY CONFORMATION_	Composite score based on linear traits below	points, 65–88	69	79.3	3.4	87
LIN_BCS_	Area between ischial tuberosities and lumbar spine	scale, 1–9	3	6.1	1.1	9
LIN_BODY_DEPTH_	Distance at last rib from spine (top) to barrel (bottom)	scale, 1–9	2	5.4	1.3	9
LIN_CHEST_WIDTH_	Width between the top of the front legs	scale, 1–9	3	6.3	1.1	9
LIN_RUMP_ANGLE_	Rump structure from hips to pins	scale, 1–9	2	4.8	0.9	7
LIN_RUMP_WIDTH_	Distance between pin bones (outer points)	scale, 1–9	3	5.7	1.2	9
LIN_STATURE_	Distance from spine (top between hips) to ground	cm, -	128	136.8	2.9	145
COM_LEGS&FEET_	Composite score based on linear traits below	points, 65–88	73	82.0	2.8	87
LIN_FOOT_ANGLE_	Angle at the front of the rear right hoof	scale, 1–9	3	5.1	1.0	8
LIN_HOCK_QUALITY_	Thickness of the ankle joint	scale, 1–9	2	6.0	1.4	9
LIN_LOCOMOTION_	Step length and straightness (movement harmony)	scale, 1–9	1	5.9	1.7	9
LIN_REAR_LEG_REAR_	Direction of feet (viewed from rear)	scale, 1–9	2	5.9	1.5	9
LIN_REAR_LEG_SIDE_	Angle at the front of the hock	scale, 1–9	3	5.3	0.9	8
COM_MAMMARY_	Composite score based on linear traits below	points, 65–88	68	78.3	3.5	87
LIN_CENTRAL_LIGAMENT_	Depth of the cleft at the base of the rear udder	scale, 1–9	1	4.3	1.4	8
LIN_FORE_UDDER_	Fore udder attachment strength to abdominal wall	scale, 1–9	2	5.3	1.3	9
LIN_FRONT_TEAT_	Front teat position (relative to udder center)	scale, 1–9	1	4.0	1.2	7
LIN_REAR_TEAT_	Rear teat position (relative to udder center)	scale, 1–9	2	5.0	1.1	8
LIN_REAR_UDDER_	Distance from vulva (bottom) to udder relative to height	scale, 1–9	1	4.3	1.4	8
LIN_TEAT_LENGTH_	Length of the front teat	scale, 1–9	2	4.9	1.0	7
LIN_UDDER_DEPTH_	Distance from udder (lowest part) to the hock	scale, 1–9	1	4.4	1.2	8

**Table 2:** Pairwise correlations between composite (COM) scores and linear (LIN) traits in DSN cows. Correlations coefficients with |*r*| ≥ 0.25 are highlighted in bold. Correlation coefficients with |*r*| ≥ 0.1 were significant after Bonferroni correction for multiple testing.

	COM_DAIRY STRENGTH_	COM_BODY CONFORMATION_	COM_LEGS&FEET_	COM_MAMMARY_
LIN_DAIRY_CHARACTER_	**0.54**	−0.06	0.10	0.05
LIN_BCS_	**−0.27**	**0.34**	0.05	0.01
LIN_BODY_DEPTH_	0.20	**0.66**	0.03	0.07
LIN_CHEST_WIDTH_	−0.05	**0.53**	0.01	0.05
LIN_RUMP_ANGLE_	−0.10	−0.06	−0.04	−0.08
LIN_RUMP_WIDTH_	0.13	**0.52**	0.05	0.05
LIN_STATURE_	**0.34**	**0.60**	0.02	0.05
LIN_FOOT_ANGLE_	0.08	0.11	**0.35**	0.10
LIN_HOCK_QUALITY_	0.13	−0.02	**0.43**	0.12
LIN_LOCOMOTION_	0.12	0.09	**0.83**	0.18
LIN_REAR_LEG_REAR_	0.15	0.11	**0.61**	0.17
LIN_REAR_LEG_SIDE_	−0.02	−0.10	**−0.30**	−0.06
LIN_CENT._LIGAMENT_	0.12	0.01	0.09	**0.36**
LIN_FORE_UDDER_	0.07	0.12	0.11	**0.53**
LIN_FRONT_TEAT_	0.11	0.11	0.15	**0.47**
LIN_REAR_TEAT_	0.11	0.13	0.07	**0.30**
LIN_REAR_UDDER_	0.20	0.06	**0.25**	**0.54**
LIN_TEAT_LENGTH_	−0.01	0.04	0.01	−0.01
LIN_UDDER_DEPTH_	0.12	0.09	0.03	**0.46**

**Table 3:** Top variants associated with conformation traits in DSN cows. Variants are sorted first by their assignment to composition scores and then by chromosomal (Chr) position. Further, rs-ID, minor allele (MA), MA frequency (MAF), β-effect of the MA (β_MA_), its standard error (SE), and the −log_10_(*p*) are provided. Corrected *p*-values were classified as highly significant (*p* < 0.01, −log_10_(*p*) = 7.76), significant (*p* < 0.05, −log_10_(*p*) = 7.06), and suggestive (*p* < 0.1, −log_10_(*p*) = 6.76). Variants belonging to loci discussed in detail in this study are highlighted in bold.

Trait/Score	Chr	Position	rs-ID	MA	MAF	β_MA_	SE(β_MA_)	−log_10_(*p*)
COM_DAIRY STRENGTH_								
**LIN_DAIRY_CHARACTER_**	**20**	**21,627,470**	**rs381893684**	**G**	**0.50**	**0.43**	**0.07**	**7.23**
COM_DAIRY STRENGTH_	X	11,548,482	-	C	0.30	−0.86	0.12	7.26
COM_BODY CONFORMATION_								
LIN_BCS_	2	109,169,387	rs378978517	C	0.16	−0.55	0.09	7.01
LIN_RUMP_ANGLE_	7	98,433,366	rs110898200	A	0.44	−0.46	0.07	6.92
LIN_RUMP_WIDTH_	8	15,021,228	rs109990310	G	0.18	−0.58	0.09	6.93
LIN_BCS_	10	90,095,068	rs385227612	ATG	0.12	0.67	0.10	6.77
**LIN_RUMP_WIDTH_**	**21**	**17,993,077**	**rs210390204**	**T**	**0.19**	**−0.58**	**0.08**	**8.27**
COM_BODY CONFORMATION_	21	37,111,947	rs132717442	A	0.15	−1.09	0.15	7.31
LIN_BCS_	24	20,358,408	rs134912642	A	0.40	0.44	0.07	7.49
**LIN_RUMP_ANGLE_**	**26**	**21,470,978**	**rs110956215**	**A**	**0.21**	**−0.55**	**0.08**	**7.24**
COM_LEGS&FEET_								
COM_LEGS&FEET_	6	105,886,046	rs133849982	C	0.19	−0.65	0.12	7.34
LIN_LOCOMOTION_	12	3,371,041	rs42662438	G	0.22	0.41	0.08	7.25
LIN_REAR_LEG_SIDE_	13	17,654,151	-	C	0.08	0.77	0.13	6.92
LIN_REAR_LEG_SIDE_	19	22,241,461	rs379037369	C	0.44	0.41	0.07	6.98
**LIN_REAR_LEG_SIDE_**	**20**	**29,327,008**	**-**	**G**	**0.38**	**0.44**	**0.07**	**7.29**
**LIN_HOCK_QUALITY_**	**23**	**28,684,391**	**rs800639948**	**GA**	**0.15**	**−0.52**	**0.09**	**6.90**
COM_LEGS&FEET_	24	10,452,692	rs110128208	T	0.09	−0.92	0.17	6.78
COM_LEGS&FEET_	26	5,538,549	rs135255414	T	0.17	−0.62	0.12	6.80
COM_MAMMARY_								
LIN_FORE_UDDER_	4	21,420,050	rs134371955	G	0.50	−0.37	0.06	7.11
LIN_REAR_UDDER_	6	15,034,445	-	T	0.05	−0.89	0.15	7.35
**LIN_CENTRAL_LIGAMENT_**	**15**	**50,014,862**	**-**	**G**	**0.38**	**0.38**	**0.06**	**7.21**
LIN_FORE_UDDER_	17	14,049,897	rs379498892	A	0.34	−0.39	0.07	7.25
LIN_CENTRAL_LIGAMENT_	18	16,330,666	rs110023445	G	0.38	−0.38	0.06	7.06
**LIN_TEAT_LENGTH_**	**22**	**17,411,573**	**rs378674848**	**T**	**0.10**	**−0.78**	**0.11**	**7.91**

**Table 4:** QTL regions and positional candidate genes for conformation traits in DSN cows. QTL regions are listed in the same order as their corresponding top variants in Table 3. QTL regions discussed in detail in this study are highlighted in bold.

**Chr**	**Position**	**QTL Start**	**QTL End**	**Positional Candidate Genes (Number of Genes)**
COM_DAIRY STRENGTH_				
**20**	**21,627,470**	**21,477,470**	**21,777,470**	**- (0)**
X	11,548,482	11,398,482	11,698,482	- (0)
COM_BODY CONFORMATION_				
2	109,169,387	109,019,387	109,319,387	- (0)
7	98,433,366	98,283,366	98,583,366	- (0)
8	15,021,228	14,871,228	15,171,228	- (0)
10	90,095,068	89,945,068	90,245,068	*NRXN3* (1)
**21**	**17,993,077**	**17,843,077**	**18,143,077**	**- (0)**
21	37,111,947	36,961,947	37,261,947	- (0)
24	20,358,408	20,208,408	20,508,408	*FHOD3*, *TPGS2*, ENSBTAG00000048612 (3)
**26**	**21,470,978**	**21,320,978**	**21,620,978**	***SEC31B*, *NDUFB8*, *HIF1AN*, *WNT8B*, *PAX2* (5)**
COM_LEGS&FEET_				
6	105,886,046	105,736,046	106,036,046	- (0)
12	3,371,041	3,221,041	3,521,041	ENSBTAG00000051623 (1)
13	17,654,151	17,504,151	17,804,151	*FBH1*, *IL15RA*, *ANKRD26*, *YME1L1*, *MASTL*, *ACBD5*, ENSBTAG00000053950 (7)
19	22,241,461	22,091,461	22,391,461	*RFLNB*, *GEMIN4*, *VPS53*, *RPH3AL*, *C19H17orf97*, *TLCD3A*, ENSBTAG00000054644 (7)
**20**	**29,327,008**	**29,177,008**	**29,477,008**	**HCN1 (1)**
**23**	**28,684,391**	**28,534,391**	**28,834,391**	***BOLA*, *TRIM10*, *TRIM15*, *BOLA*-*NC1*, *JSP.1*, *TRIM26*, *TRIM40*, ENSBTAG00000037421 (8)**
24	10,452,692	10,302,692	10,602,692	- (0)
26	5,538,549	5,388,549	5,688,549	*PCDH15* (1)
COM_MAMMARY_				
4	21,420,050	21,270,050	22,207,653	*ETV1* (1)
6	15,034,445	14,884,445	15,184,445	*ENPEP*, ENSBTAG00000054468 (2)
**15**	**50,014,862**	**49,373,191**	**50,164,862**	***OR51AB4*, *OR52AB4*, *OR52AE11*, *OR51G4*, *OR52J3D*, ENSBTAG00000038578, *OR51AG1*, ENSBTAG00000039185, *OR51P1*, *OR51V22*, *OR52Z12*, *OR52AB9*, *OR52E58*, *OR52S27*, *OR52Z19*, *OR52AD1*, *OR52A24*, *OR51L15*, *OR51G2*, *OR51L1B*, ENSBTAG00000049986, ENSBTAG00000050365, *OR51V1B*, ENSBTAG00000051323, *OR52J1*, *OR52S6*, *OR52E1*, *OR51S1B*, *OR51P1B*, *OR51L22*, *OR51L1C*, *OR52S20*, *OR51A8*, *OR51H5* (34)**
17	14,049,897	13,899,897	14,281,310	*GYPA*, *GYPB* (2)
18	16,330,666	16,180,666	16,480,666	- (0)
**22**	**17,411,573**	**17,261,573**	**17,561,573**	***SRGAP3* (1)**

## Data Availability

The datasets analyzed in this study can be found in the European Nucleotide Archive (ENA) (https://www.ebi.ac.uk/ena/browser/home, accessed on 5 February 2025) (WGS data: PRJEB45822, DSN200k SNP chip data: PRJEB46861, IlluminaBovineSNP50 BeadChip data: PRJEB42513).

## Supplementary Materials

The following supporting information can be downloaded at https://www.mdpi.com/article/10.3390/genes16040445/s1, Table S1: Selection of fixed effects for GWAS models using Akaike information criterion (AIC); Table S2: Inflation factors λ from GWAS results for the investigated conformation traits; Table S3: Full list of associated variants (*p* < 0.1) from GWAS with conformation traits in DSN cows; Table S4: List of sequence variant impacts in QTL regions based on Ensembl VEP annotation; Table S5: Functional enrichment analysis of sequence variants in QTL regions using g:Profiler; Table S6: List of publications with GWAS results overlap with loci identified in this study.

## References

[1] Bundesanstalt für Landwirtschaft und Ernährung (BLE) (2023). Einheimische Nutztierrassen in Deutschland Und Rote Liste Gefährdeter Nutztierrassen 2023.

[2] Adler B., Gassan M., Thiele M. (2023). Deutsches Schwarzbuntes Niederungsrind—50 Jahre DSN-Genreservezucht in Brandenburg.

[3] Rinderzuchtverband Berlin-Brandenburg eG (RZB eG) (2022). Zuchtprogramm Für Die Rasse Deutsches Schwarzbuntes Niederungsrind.

[4] Pausch H., Emmerling R., Schwarzenbacher H., Fries R. (2016). A Multi-Trait Meta-Analysis with Imputed Sequence Variants Reveals Twelve QTL for Mammary Gland Morphology in Fleckvieh Cattle. Genet. Sel. Evol. GSE.

[5] Wu X., Fang M., Liu L., Wang S., Liu J., Ding X., Zhang S., Zhang Q., Zhang Y., Qiao L. (2013). Genome Wide Association Studies for Body Conformation Traits in the Chinese Holstein Cattle Population. BMC Genom..

[6] Flury C., Boschung C., Denzler M., Bapst B., Schnyder U., Gredler B., Signer-Hasler H. Genome-wide association study for 13 udder traits from linear type classification in cattle. Proceedings of the 10th World Congress on Genetics Applied to Livestock Production.

[7] Nazar M., Abdalla I.M., Chen Z., Ullah N., Liang Y., Chu S., Xu T., Mao Y., Yang Z., Lu X. (2022). Genome-Wide Association Study for Udder Conformation Traits in Chinese Holstein Cattle. Animals.

[8] Wolf M.J., Yin T., Neumann G.B., Korkuć P., Brockmann G.A., König S., May K. (2021). Genome-Wide Association Study Using Whole-Genome Sequence Data for Fertility, Health Indicator, and Endoparasite Infection Traits in German Black Pied Cattle. Genes.

[9] May K., Scheper C., Brügemann K., Yin T., Strube C., Korkuć P., Brockmann G.A., König S. (2019). Genome-Wide Associations and Functional Gene Analyses for Endoparasite Resistance in an Endangered Population of Native German Black Pied Cattle. BMC Genom..

[10] Korkuć P., Arends D., May K., König S., Brockmann G.A. (2021). Genomic Loci Affecting Milk Production in German Black Pied Cattle (DSN). Front. Genet..

[11] Korkuć P., Neumann G.B., Hesse D., Arends D., Reißmann M., Rahmatalla S., May K., Wolf M.J., König S., Brockmann G.A. (2023). Whole-Genome Sequencing Data Reveal New Loci Affecting Milk Production in German Black Pied Cattle (DSN). Genes.

[12] Meier S., Arends D., Korkuć P., Neumann G.B., Brockmann G.A. (2020). A Genome-Wide Association Study for Clinical Mastitis in the Dual-Purpose German Black Pied Cattle Breed. J. Dairy Sci..

[13] Neumann G.B., Korkuć P., Arends D., Wolf M.J., May K., Reißmann M., Elzaki S., König S., Brockmann G.A. (2021). Design and Performance of a Bovine 200 k SNP Chip Developed for Endangered German Black Pied Cattle (DSN). BMC Genom..

[14] Browning B.L., Zhou Y., Browning S.R. (2018). A One-Penny Imputed Genome from next-Generation Reference Panels. Am. J. Hum. Genet..

[15] Danecek P., Auton A., Abecasis G., Albers C.A., Banks E., DePristo M.A., Handsaker R.E., Lunter G., Marth G.T., Sherry S.T. (2011). The Variant Call Format and VCFtools. Bioinformatics.

[16] Rosen B.D., Bickhart D.M., Schnabel R.D., Koren S., Elsik C.G., Zimin A., Dreischer C., Schultheiss S., Hall R., Schroeder S.G. Modernizing the Bovine Reference Genome Assembly. Proceedings of the 11th World Congress on Genetics Applied to Livestock Production.

[17] Christensen R.H.B. Ordinal—Regression Models for Ordinal Data. R Package Version 2023.12-4.1, 2023. https://cran.r-project.org/web/packages/ordinal/index.html.

[18] Venables W.N., Ripley B.D. (2002). Modern Applied Statistics with S.

[19] Purcell S., Neale B., Todd-Brown K., Thomas L., Ferreira M.A.R., Bender D., Maller J., Sklar P., De Bakker P.I.W., Daly M.J. (2007). PLINK: A Tool Set for Whole-Genome Association and Population-Based Linkage Analyses. Am. J. Hum. Genet..

[20] Martin F.J., Amode M.R., Aneja A., Austine-Orimoloye O., Azov A.G., Barnes I., Becker A., Bennett R., Berry A., Bhai J. (2023). Ensembl 2023. Nucleic Acids Res..

[21] Raudvere U., Kolberg L., Kuzmin I., Arak T., Adler P., Peterson H., Vilo J. (2019). G:Profiler: A Web Server for Functional Enrichment Analysis and Conversions of Gene Lists (2019 Update). Nucleic Acids Res..

[22] McLaren W., Gil L., Hunt S.E., Riat H.S., Ritchie G.R.S.S., Thormann A., Flicek P., Cunningham F. (2016). The Ensembl Variant Effect Predictor. Genome Biol..

[23] Hu Z.L., Park C.A., Reecy J.M. (2023). A Combinatorial Approach Implementing New Database Structures to Facilitate Practical Data Curation Management of QTL, Association, Correlation and Heritability Data on Trait Variants. Database.

[24] Fantini D. EasyPubMed: Search and Retrieve Scientific Publication Records from PubMed. R Package Version 2.13, 2019. https://10.32614/CRAN.package.easyPubMed.

[25] R Core Team (2023). R: A Language and Environment for Statistical Computing.

[26] Wickham H. (2016). ggplot2: Elegant Graphics for Data Analysis.

[27] Wickham H. (2007). Reshaping Data with the Reshape Package. J. Stat. Softw..

[28] Kassambara A. ggpubr: “ggplot2” Based Publication Ready Plots. R Package Version 0.6.0, 2020. https://10.32614/CRAN.package.ggpubr.

[29] Loker S., Bastin C., Miglior F., Sewalem A., Schaeffer L.R., Jamrozik J., Ali A., Osborne V. (2012). Genetic and Environmental Relationships between Body Condition Score and Milk Production Traits in Canadian Holsteins. J. Dairy Sci..

[30] Khmelnychyii L., Khmelnychyii S., Samokhina Y. (2023). Correlation between Descriptive and Group Type Traits in the System of Cow’s Linear Classification of Ukrainian Brown Dairy Breed. Open Agric..

[31] Marete A.G., Guldbrandtsen B., Lund M.S., Fritz S., Sahana G., Boichard D. (2018). A Meta-Analysis Including Pre-Selected Sequence Variants Associated with Seven Traits in Three French Dairy Cattle Populations. Front. Genet..

[32] Bouwman A.C., Daetwyler H.D., Chamberlain A.J., Ponce C.H., Sargolzaei M., Schenkel F.S., Sahana G., Govignon-Gion A., Boitard S., Dolezal M. (2018). Meta-Analysis of Genome-Wide Association Studies for Cattle Stature Identifies Common Genes That Regulate Body Size in Mammals. Nat. Genet..

[33] Cole J.B., Wiggans G.R., Ma L., Sonstegard T.S., Lawlor T.J., Crooker B.A., Van Tassell C.P., Yang J., Wang S., Matukumalli L.K. (2011). Genome-Wide Association Analysis of Thirty One Production, Health, Reproduction and Body Conformation Traits in Contemporary U.S. Holstein Cows. BMC Genom..

[34] Cohen-Zinder M., Asher A., Lipkin E., Feingersch R., Agmon R., Karasik D., Brosh A., Shabtay A. (2016). FABP4 Is a Leading Candidate Gene Associated with Residual Feed Intake in Growing Holstein Calves. Physiol. Genom..

[35] Snelling W.M., Allan M.F., Keele J.W., Kuehn L.A., McDaneld T., Smith T.P.L., Sonstegard T.S., Thallman R.M., Bennett G.L. (2010). Genome-Wide Association Study of Growth in Crossbred Beef Cattle. J. Anim. Sci..

[36] Lu D., Miller S., Sargolzaei M., Kelly M., Vander Voort G., Caldwell T., Wang Z., Plastow G., Moore S. (2013). Genome-Wide Association Analyses for Growth and Feed Efficiency Traits in Beef Cattle1. J. Anim. Sci..

[37] Kolbehdari D., Wang Z., Grant J.R.R., Murdoch B., Prasad A., Xiu Z., Marques E., Stothard P., Moore S.S.S. (2008). A Whole-Genome Scan to Map Quantitative Trait Loci for Conformation and Functional Traits in Canadian Holstein Bulls. J. Dairy Sci..

[38] Igoshin A.V., Yudin N.S., Romashov G.A., Larkin D.M. (2023). A Multibreed Genome-Wide Association Study for Cattle Leukocyte Telomere Length. Genes.

[39] Do D.N., Bissonnette N., Lacasse P., Miglior F., Sargolzaei M., Zhao X., Ibeagha-Awemu E.M. (2017). Genome-Wide Association Analysis and Pathways Enrichment for Lactation Persistency in Canadian Holstein Cattle. J. Dairy Sci..

[40] Babiuk S., Horseman B., Zhang C., Bickis M., Kusalik A., Schook L.B., Abrahamsen M.S., Pontarollo R. (2007). BoLA Class I Allele Diversity and Polymorphism in a Herd of Cattle. Immunogenetics.

[41] Chen S.Y., Oliveira H.R., Schenkel F.S., Pedrosa V.B., Melka M.G., Brito L.F. (2020). Using Imputed Whole-Genome Sequence Variants to Uncover Candidate Mutations and Genes Affecting Milking Speed and Temperament in Holstein Cattle. J. Dairy Sci..

[42] Tijjani A., Utsunomiya Y.T., Ezekwe A.G., Nashiru O., Hanotte O. (2019). Genome Sequence Analysis Reveals Selection Signatures in Endangered Trypanotolerant West African Muturu Cattle. Front. Genet..

[43] Connor E.E., Zhou Y., Liu G.E. (2018). The Essence of Appetite: Does Olfactory Receptor Variation Play a Role?. J. Anim. Sci..

[44] Wu C., Jia Y., Lee J.H., Kim Y., Sekharan S., Batista V.S., Lee S.J. (2015). Activation of OR1A1 Suppresses PPAR-γ Expression by Inducing HES-1 in Cultured Hepatocytes. Int. J. Biochem. Cell Biol..

[45] An S.S., Liggett S.B. (2018). Taste and Smell GPCRs in the Lung: Evidence for a Previously Unrecognized Widespread Chemosensory System. Cell. Signal..

[46] Kang N., Bahk Y.Y., Lee N., Jae Y., Cho Y.H., Ku C.R., Byun Y., Lee E.J., Kim M.S., Koo J. (2015). Olfactory Receptor Olfr544 Responding to Azelaic Acid Regulates Glucagon Secretion in α-Cells of Mouse Pancreatic Islets. Biochem. Biophys. Res. Commun..

[47] Krause F., Mohebian K., Delpero M., Hesse D., Kühn R., Arends D., Brockmann G.A. (2022). A Deletion Containing a CTCF-Element in Intron 8 of the Bbs7 Gene Is Partially Responsible for Juvenile Obesity in the Berlin Fat Mouse. Mamm. Genome.

[48] Mattick J.S., Makunin I.V. (2006). Non-Coding RNA. Hum. Mol. Genet..

